# The Isolation and Identification of *Pseudoalteromonas* sp. H27, a Bacterial Strain Pathogenic to *Crassostrea gigas*

**DOI:** 10.3390/microorganisms13020296

**Published:** 2025-01-30

**Authors:** Heyang Qin, Junyi Jiang, Zhikai Jing, Jiayu Wang, Shuang Xu, Rongwei Chen, Bo Wang, Zhongming Huo, Lei Fang

**Affiliations:** 1 College of Marine Science and Environment, Dalian Ocean University, Dalian 116023, China; 16643333659@163.com (H.Q.); 17280802536@163.com (J.J.); 18153985685@163.com (Z.J.); 18242450575@163.com (J.W.); 13263368672@163.com (S.X.); 18305911928@163.com (R.C.); wangbo_980110@163.com (B.W.); 2 College of Fisheries and Life Science, Dalian Ocean University, Dalian 116023, China; 3 Engineering Research Center of Shellfish Culture and Breeding, Dalian Ocean University, Dalian 116023, China

**Keywords:** *C. gigas*, bacteria, pathogenic, histopathological analysis, bacterial challenge experiment, *Pseudoalteromonas*

## Abstract

Bacterial infection is frequently observed in disease outbreaks of aquatic animals, making it of significance to isolate and identify the bacterial pathogens. In this study, diseased individuals of *Crassostrea gigas* were sampled from the nearshore area in Zhanjiang, Guangdong in May 2023. Culturable bacteria were isolated from the diseased tissue and a pathogenic strain labeled as H27 was screened through a hemolysis test and bacterial challenge experiments. Morphological characterization, 16S rRNA gene sequence-based molecular identification and biochemical tests showed that strain H27 belonged to the genus of *Pseudoalteromonas*, a dominant genus in the diseased tissue of *C. gigas* revealed by bacterial community structure analysis. The clinical signs originally observed in naturally diseased *C. gigas* were reproduced in strain H27-challenged adults, both with the red mantle and adductor. Histopathological analysis was further performed on the diseased tissues of the latter, which showed a significantly increased accumulation of pigment granules in the cytoplasm of the diseased mantle as well as enlarged muscle fiber distances in the diseased adductor. In addition, strain H27 was re-isolated from tissues of the moribund *C. gigas* after bacterial challenge, indicating the fulfillment of Koch’s postulate. Our results help to enrich the knowledge of *C. gigas* diseases, possibly contributing to disease prevention and control.

## 1. Introduction

Oysters are traditionally cultured shellfish with a huge production and high economic value. According to the statistics released by the FAO (Food and Agriculture Organization of the United Nations) in 2024, global production of oyster aquaculture reached 58.5 million tonnes in 2021, accounting for 16.9% of total aquaculture production. The production value of maricultured oysters reached USD 631.5 million, accounting for 5.4% of the total production value of aquaculture [1]. In China, the culture area of oysters in 2023 reached 276,816 hectares, with a 17.82% increment in comparison with the previous year [2]. *Crassostrea gigas*, also known as the Pacific Oyster, belongs to Mollusca, Bivalvia, Ostreidam, Ostreidae and *Crassostrea* [3]. It is widely cultured in China due to its strong reproductive capacity, fast growth rate and high nutritional value [4]. However, cultured *C. gigas* have been suffering from frequent diseases in recent years, caused by both abiotic factors and biotic factors. Abnormal climate and the deterioration of water quality are major abiotic factors, whereas pathogenic viruses, bacteria, fungi and parasites are common biotic factors. Bacterial diseases often occur in outbreaks with high mortality. *Vibrio anguillarum*, *V. splendidus* and *Pseudomonas fluorescens* have been reported to be bacterial pathogens of *C. gigas* [5,6]. Human pathogenic *Vibrio* species have been associated with gastroenteritis (*V. parahaemolyticus* and *V. cholerae* non-O1/O139) after the consumption of contaminated food [7]. According to regulations governing the sanitary control of shellfish, all bacterial disease outbreaks must undergo epidemiological studies to ensure that precise measures can be taken to prevent further infections [8]. *Arcobacter*, *Marinobacterium*, *Marinomonas*, *Vibrio* and *Pseudoalteromonas* are associated with Pacific Oyster Mortality Syndrome (POMS) and are collectively termed as the “POMS pathobiota” [9]. Nevertheless, it remains unknown whether other lethal bacterial flora are involved and how these bacterial communities interact with each other [10]. Functional complementarity could partly explain it, which states that competition for limited resources among the pathobiota allows for efficient utilization of diverse resources in oysters, making the coexistence of bacteria with distinct metabolic characteristics possible [11].

Bacteria of the *Pseudoalteromonas* genus are aerobic or facultatively anaerobic, tending to move through a single sheathless polar flagella [12]. They are widely distributed in marine environments [13,14]. Previous findings have shown that members of *Pseudoalteromonas* could secrete extracellular enzymes leading to algae lysis [15,16] or produce toxins lethal to *Penaeus japonicus* [17] and *Cancer pagurus* [18]. *Pseudoalteromonas* could also directly infect marine organisms, causing green spot disease [19,20] and yellow spot disease [21] in *Pyropia yezoensis*, red spot disease [22] and spot-wounded disease [23] in *Laminaria japonica*, stomach ulcer disease [24] and skin ulcerative syndrome in *Apostichopus japonicas* [25,26,27], syndrome disease in *Strongylocentrotus intermedius* [28], etc. In terms of the *Pseudoalteromonas*-conferred infection in *Acanthopagrus schlegel* [29], *Epinephelus septemfasciatus* Thunberg [30], *Cynoglossus semilaevis* Günther [31] and *Dicentrarchus labrax* L. [32], epidermis and fin ulceration are frequently observed, with death occurring occasionally. In the case of *C. gigas* mass death in summer, bacteria of the *Pseudoalteromonas* genus were often isolated as the dominant species from the diseased tissues [33,34]. Nevertheless, it is rarely reported that *Pseudoalteromonas* can directly cause diseases in *C. gigas*.

In May 2023, there was a disease outbreak in *C. gigas* cultured in the coastal areas of Zhanjiang, Guangdong, China, with red tissues observed in the diseased individuals. In this study, the diseased *C. gigas* were used as the experimental material and pathological bacteria were isolated from the diseased tissues. Morphological characterization, 16S rRNA gene sequence analysis, biochemical tests and bacterial challenge experiments showed that a bacterial strain, H27, was pathogenic to *C. gigas* and belonged to the genus of *Pseudoalteromonas*.

## 2. Materials and Methods

### 2.1. Sampling of Crassostrea gigas

The infected individuals of *C. gigas* were collected from the aquaculture area located in Zhanjiang, Guangdong Province, China in May 2023, where the mass infection of *C. gigas* was observed. Healthy individuals of *C. gigas* cultured in the adjacent area were collected as well. The collected oysters were transported on ice to Dalian Ocean University (Dalian, Liaoning Province, China) via flight (<24 h). The healthy *C. gigas* larvae and adults used for the bacterial challenge experiment were collected from Shicheng Island, Zhuanghe, Dalian, and were temporarily cultured at Zhangzidao Co., Ltd. in Dalian, China; these were of the same species as the diseased *C. gigas*. The larvae retained on the 100 μm sieve were rinsed with sterile seawater with a salinity of 28 to 30 psu and a temperature of 21 °C to 23 °C. Then, the larvae were placed in a container filled with sterile seawater for temporary culture. Both the larvae and the adults were temporarily cultured for 7 days at room temperature (21 °C to 23 °C), during which they were fed with microalgae three times a day, with continuous areation and a daily water change. They were inspected once a day and dead individuals were removed.

### 2.2. Analysis of the Bacterial Community Structure in the Soft Tissue of C. gigas

To analyze the bacterial community structure, soft tissues of the diseased *C. gigas* and the healthy *C. gigas* samples were collected using a sterile scalpel. The total DNA of the tissues was extracted using the CTAB method [35]. The V4 region of the bacterial 16S rRNA gene was amplified using 515F (5′-GTGCCAGCMGCCGCGGTAA-3′) and 806R (5′-GGACTACHVGGGTWTCTAAT-3′) primers. The amplicons were sequenced using the Illumina NovaSeq PE250 high-throughput sequencing platform (BGI). Mothur package (v. 1.31.2) [36] was used to select the unique tag. The standard of the sequence similarity was set at 97% to classify the operational taxonomy units (OTUs). QIIME (v. 1.8.0) was used to analyze α-diversity (Shannon index and Chao1 index) and Pheatmap (R software v.4.0.3) was used to analyze β-diversity.

### 2.3. Isolation of Culturable Bacteria from Diseased Tissue of C. gigas

The infected individuals of *C. gigas* were dissected using a sterile scalpel and red-colored tissue was identified as the diseased tissue. A sterile pestle was used to homogenize the tissue in seawater. The samples were plated on Zobell Marine Broth 2216 (2216E) (Qingdao Haibo Biotechnology Co., Ltd., Qingdao, China) agar plates (pH 7.6 ± 0.2) and then aerobically incubated at 30 °C in a constant temperature incubator. The dominant colonies with the same morphology were picked and purified via plate streaking at least three times.

### 2.4. Hemolysis Test

The dominant bacterial strain isolated from the diseased tissues of *C. gigas* was inoculated into Zobell Marine Broth 2216 liquid medium (pH 7.6 ± 0.2) (Qingdao Haibo Biotechnology Co., Ltd., Qingdao, China) and cultured at 30 °C, 120 rpm for 24 h. The composition of the 2216E medium includes 5.0 g/L of peptones, 1.0 g/L of yeast extract, 0.1 g/L of ferric citrate, 19.45 g/L of sodium chloride, 5.98 g/L of magnesium chloride, 3.24 g/L of sodium sulfate, 1.8 g/L of calcium chloride, 0.55 g/L of potassium chloride, 0.16 g/L of sodium carbonate, 0.08 g/L of potassium bromide, 0.034 g/L of strontium chloride, 0.022 g/L of boric acid, 0.004 g/L of sodium silicate, 0.0024 g/L of sodium fluoride, 0.0016 g/L of ammonium nitrate and 0.008 g/L of disodium phosphate. A total of 2.5 μL of the liquid culture was spotted on a Columbia blood agar plate (Qingdao Hi-Tech Industrial Park Hope Bio-Technology Co., Ltd., Qingdao, China). When the liquid culture was completely absorbed, the plate was incubated upside down at 30° C in a constant temperature incubator for one week and examined every 24 h to observe whether there was a β-hemolysis ring.

### 2.5. Identification of Bacterial Strains

#### 2.5.1. Morphological Characterization

The dominant bacterial strain was inoculated onto a 2216E agar plate via plate streaking and then incubated upside down at 30 °C for 24 h. The morphological characteristics of the colonies were observed. Furthermore, the pure culture was taken and plated onto sterile double distilled water on a clean glass slide and subjected to Gram staining using a Gram staining kit (G-clone, cat. NO.RS3140, Beijing, China) following the instructions provided by the manufacturer. The morphology of the bacterial cells was observed under a microscope using an oil immersion lens.

#### 2.5.2. Molecular Identification Based on the Full-Length Sequence of 16S rRNA Gene

For molecular identification of the bacterial strain, the total DNA was extracted from the pure culture using a bacterial DNA extraction kit according to the manufacturer’s instructions (Tiangen, Beijing, China). The molecular identification of the tested strain was performed based on the full-length sequence of the 16S rRNA gene. The full length of the 16S rRNA gene was amplified using the universal primer pair 27F (5′-AGAGTTTGATCCTGGCTCAG-3′) and 1492R (5′-GGTTACCTTGTTACGACTT-3′) [37]. The PCR products were sent to Beijing Genomics Institute (Beijing, China) for Sanger sequencing and the gene sequence was subject to a BLAST search against the gene database in the NCBI (National Center for Biotechnology Information) website to identify the strain to the genus level. The Neighbor-Joining method of MEGA 11.0 was used to construct the phylogenetic tree according to the 16S rRNA gene sequences from different species in the same genus.

#### 2.5.3. Metabolic Profiling and Antibiotics Sensitivity Analysis

The bacterial strain was metabolically profiled using a Biolog Gen III Microplate (Guangzhou Hua Yue Instrument Co., Ltd., Guangzhou, China) as previously described [38]. The wells were compared to Biolog’s species database library using Biolog’s Microbial Identification Systems software (GEN III Retrospect 2.0 Data Management Software). When cultured for 16–24 h, if the similarity (SIM) value is ≥0.50, the system automatically provides the identification results to the species level.

The antibiotic sensitivity of the bacterial strain was determined using the Kirby–Bauer disk diffusion method. The strain was inoculated into 2216E liquid medium and shaking cultured at 30 °C at 120 rpm for 24 h. The liquid culture was adjusted to a final concentration of 1 × 10^7^ CFU/mL using fresh 2216E liquid medium and 100 μL of the diluted liquid culture was plated onto 2216E agar plates. Test papers (purchased from Hangzhou Binhe Microbiology Reagent Co., Hangzhou, China) containing different antibiotics were placed on the above-mentioned agar plates plated with the liquid culture. The agar plates were incubated at 30 °C for 24 h and the diameters of the inhibition zones which appeared were measured using Vernier calipers. The strain’s sensitivity to different antibiotics was described as sensitive (S), moderately sensitive (M) or resistant (R) according to the Clinical and Laboratory Standards Institute (CLSI) guidelines.

### 2.6. Bacterial Challenge Experiment

**Figure 1:**
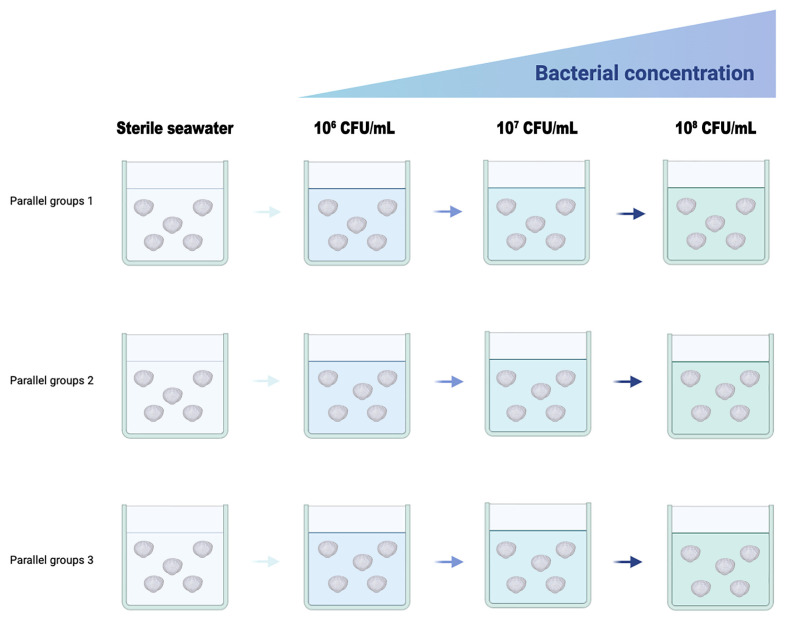
Scheme of the bacterial challenge experiment using larval *C. gigas*.

For the bacterial strain subject to bacterial challenge experiments, the larval *C. gigas* were involved using the dipping method (Figure 1). The larvae were first suspended in sterile seawater and then randomly divided into four groups with a density of 4–6 larvae/mL. Among the four groups, one was set as the control group and three were set as the treatment groups. For each group, three individual replicates were set. The larvae were cultured in filtered and sterilized sea water at 21–23 °C with continuous areation and fed with microalgae three times a day. A complete water refill was carried out once a day, right before adding the bacteria culture. For the three treatment groups, a liquid culture of the bacteria was added to final concentrations of 1 × 10^6^ CFU/mL, 1 × 10^7^ CFU/mL and 1 × 10^8^ CFU/mL [39,40], respectively. The larvae were observed and counted under a dissecting microscope every 24 h during the 5 days experiment period.

### 2.7. Histopathological Observation

To observe the pathological changes in the tissues of adult *C. gigas* caused by the pathogenic strain, a bacterial challenge experiment using the injection method was conducted. Individual adults were divided into two groups, the control group and the treatment group, with 6 individuals in each group. For the control group, 0.1 mL of sterile seawater was injected into the mantle using a sterile injector. For the treatment group, 0.1 mL of sterile seawater containing 1.94 × 10^5^ CFU/g of bacteria was injected into the mantle using a sterile injector. The moribund individuals of challenged *C. gigas* were dissected and the tissues were collected for subsequent histopathological observation. The healthy *C. gigas* in the control group were retained for daily observation and normal culturing. For histopathological analysis of adult *C. gigas* tissues, the control samples were taken on day 7 after injection with sterile seawater. The control group and the challenge group both included samples of adductor muscle, mantle, digestive gland, gills and gonads. For both the healthy *C. gigas* from the control group and the moribund *C. gigas* from the challenge group, the samples were collected and fixed with 4% polyformaldehyde solutions. The fixed specimens were then subjected to paraffin embedding, hematoxylin and eosin (H&E) staining, neutral resin sealing and sectioning into 4 μm thick sections. The sections were observed under an optical microscope (Olympus, Tokyo, Japan) to identify the histopathological changes of tissues from the challenge group as compared with the control group.

### 2.8. Data Processing and Analysis

The survival rate of the larvae was calculated using the following formula: survival rate = number of survived larvae/initial number of larvae × 100%. Data were analyzed using one-way analysis of variance (ANOVA) in SPSS software (version 26). Significance was assessed using the Duncan multiple range test, and difference was considered significant at *p* < 0.01.

## 3. Results

### 3.1. Clinical Signs of Naturally Diseased C. gigas

**Figure 2:**
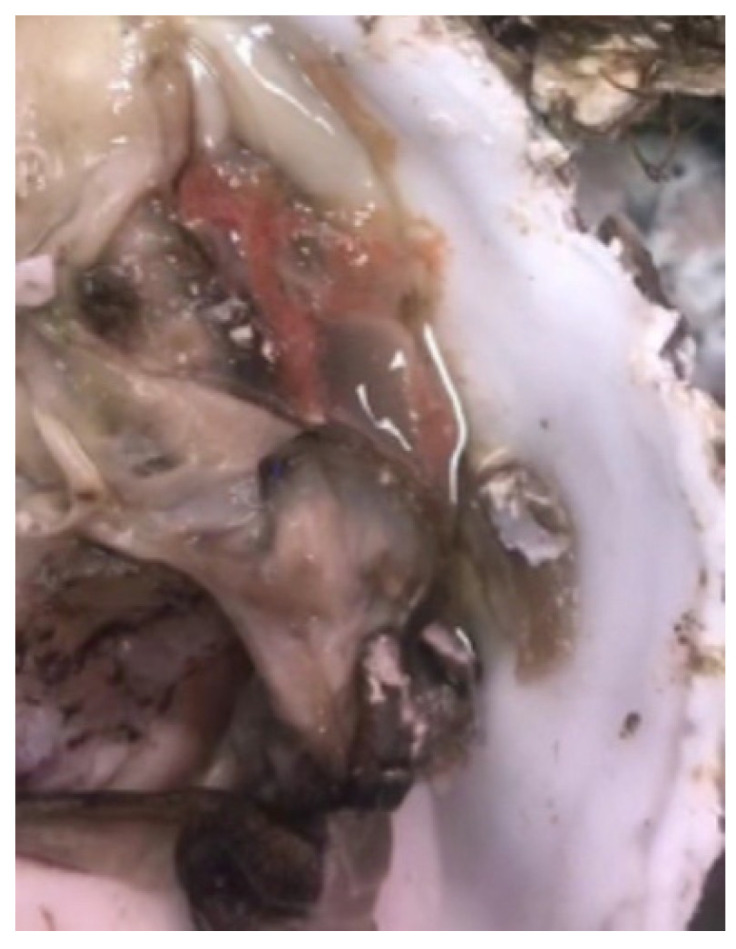
The clinical signs of the diseased *C. gigas*.

The diseased *C. gigas* showed decreased activity, with an average weight of 44.0 ± 7.0 g. They were dissected and the soft tissues were observed, as shown in Figure 2. The mantle of the diseased *C. gigas* was atrophied and turned red, with weakened adductor muscle contraction and a delay in response to external stimuli.

### 3.2. Bacterial Community Structure Analysis of the Soft Tissues in C. gigas

**Figure 3:**
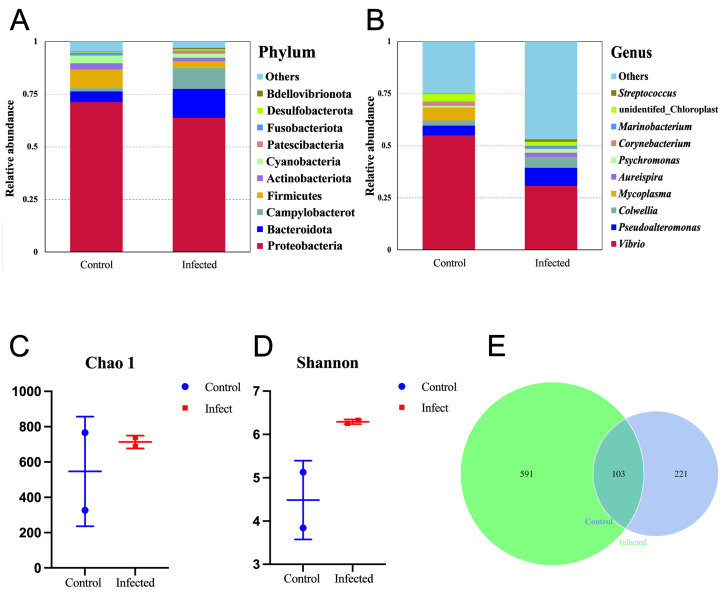
Diversity analysis of bacteria in *C. gigas* soft tissues. (**A**) The phylum level. (**B**) The genus level. (**C**) Chao 1. (**D**) Shannon. (**E**) OTU abundance Venn diagram analysis of healthy and diseased *C. gigas*.

Taxonomic compositions at the phylum level of the bacterial communities are shown in Figure 3A. For both the healthy and the infected *C. gigas*, Proteobacteria held the overwhelming predominance, with a relative abundance above 60%. However, the community structures of these two groups differed from each other in terms of the other dominant phyla. In the healthy *C. gigas*, the other dominant phyla in a descending order were Firmicutes, Bacteroidota, Cyanobacteria, Actinobacteriota, etc. In the infected *C. gigas*, the other dominant phyla in a descending order were Bacteroidota, Campylobacterota, Firmicutes, Actinobacteriota, Cyanobacteria, etc.

Taxonomic compositions at the genus level of the bacterial communities are shown in Figure 3B. Compared with the phylum level, the difference in the bacterial community structures of the two groups at the genus level was more significant. For both groups, *Vibrio* was the most predominant genus, with a relative abundance of 54.9% in the healthy *C. gigas* and a relative abundance of 30.6% in the infected *C. gigas*. In the healthy *C. gigas*, the other dominant genera in a descending order were *Mycoplasma* (6.3%), *Pseudoalteromonas* (4.8%), *Colwellia* (2.4%), etc. However, in the infected *C. gigas*, the other dominant genera in a descending order were *Pseudoalteromonas* (8.7%), *Colwellia* (4.6%), *Aureispira* (2.3%), etc. The abundance of *Pseudoalteromonas* in the infected *C. gigas* increased as compared with the healthy *C. gigas*.

Analysis of the α-diversity was performed and the results are shown in Figure 3C,D. The Chao1 and Shannon indexes of infected *C. gigas* were higher than those of healthy *C. gigas*, yet the difference was not significant (*p* > 0.05). In terms of the number of OTUs, 694 were detected from the infected group and 324 were detected from the healthy group (Figure 3E). In addition, the two groups shared 103 identical OTUs.

### 3.3. Isolation of Culturable Bacteria from Diseased C. gigas Tissues

For isolating culturable bacteria from diseased *C. gigas* tissues, the dominant colonies with the same morphology and color were picked and purified at least three times. The isolated strain was named as H27.

### 3.4. Hemolytic Detection of Strain H27

**Figure 4:**
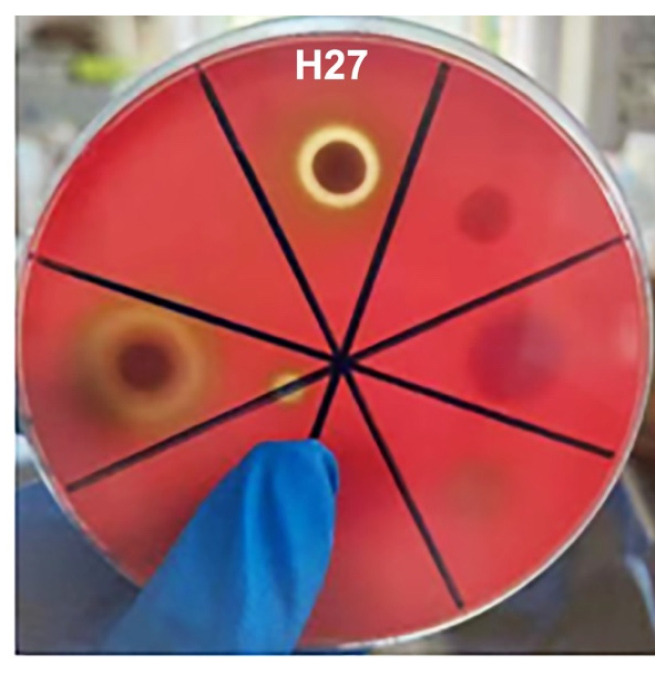
Hemolysis test of culturable bacteria. Strain H27 produced a clear transparent ring around the colony.

A hemolytic test showed that strain H27 produced a clear transparent ring around the colony (Figure 4), suggesting that it could produce a β-hemolysis ring.

### 3.5. Morphological Characterization and Molecular Identification of Strain H27

**Figure 5:**
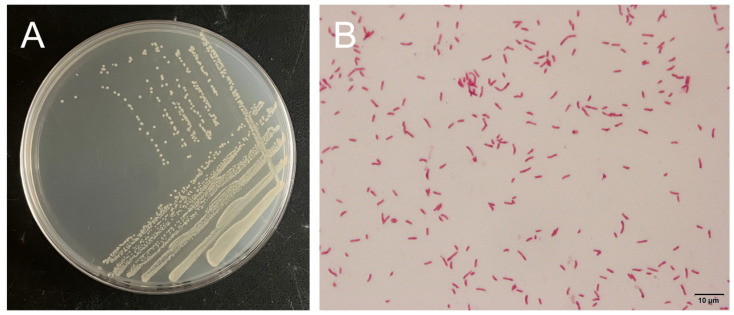
Morphological characteristics of strain H27. (**A**) Colonies of strain H27 were round, smooth and light yellow on 2216E agar plate. (**B**) Microscopic image of Gram-stained strain H27 was red (magnification: 100×).

Colonies of strain H27 on 2216E agar plate had a round shape and a smooth surface with an opaque light-yellow color, as shown in Figure 5A. Under the microscope, the Gram-stained cells of strain H27 were red colored and had a single/double rod shape, suggesting that it was a Gram negative rod bacterium (Figure 5B).

**Figure 6:**
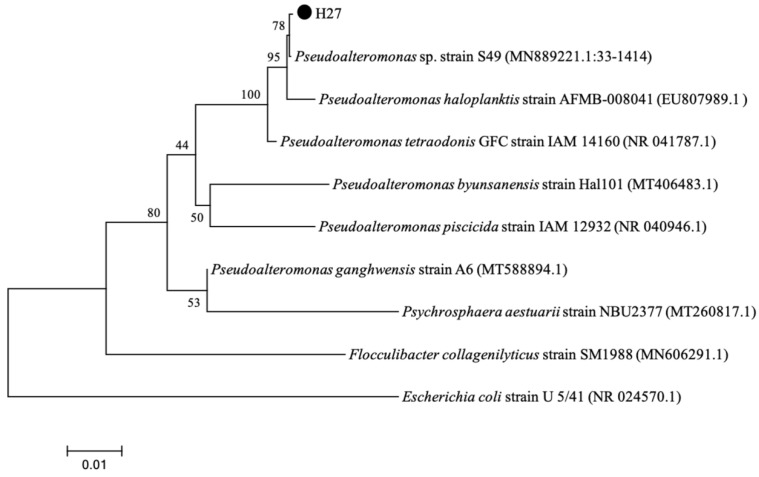
Phylogenetic tree of strain H27 based on 16S rRNA gene sequences. According to the bootstrapping data (1000 replications), values at the node indicate the percentage of trees which fall into this grouping. The scale bar indicates the number of substitutions per site. The phylogenetic tree showed that strain H27 was clustered with *P*. sp. strain S49 (MN889221.1).

The results showed that strain H27 was most similar to *Pseudoalteromonas* sp. strain S49, with 99.93% of identity under 100% of coverage. As shown in Figure 6, strain H27 (PQ113772) clustered with *P.* sp. strain S49 and formed an individual branch. The results of morphological characterization and molecular identification both implied that strain H27 belonged to the genus of *Pseudoalteromonas*.

### 3.6. Metabolic Profiling and Antibiotic Sensitivity Analysis of Strain H27

The metabolic profiles of strain H27 using a Biolog Gen III Microplate are shown in Table 1. The results showed that strain H27 could grow at pH 6, 1–8% NaCl. The carbon sources it could utilize included dextrin, D-maltose, L-arginine, L-serine, N-acetyl-D-glucosamine, α-D-glucose, D-fructose, D-fructose-6-PO_4_, acetic acid, propionic acid, L-alanine, L-glutamic acid, inosine, D-galactose, L-malic acid, D-mannitol, D-glucose-6-PO_4_, D-galacturonic acid, L-galactonic acid lactone, D-gluconic acid, D-glucuronic acid, gelatin, glucuronamide, N-acetyl-β-D-mannosamine, N-acetyl neuraminic acid, acetoacetic acid and glycyl-L-prolineas. Additionally, it could grow in the presence of vancomycin, tetrazolium violet, 1% sodium lactate, fusidicacid, potassium tellurite and aztreonam. A search against the Biolog Gen III database revealed that strain H27 was most similar to *Neisseria flava*, *Streptococcus halichoeri*, *Virgibacillus halodenitrificans* and *Tsukamurella paurometabola*, with an SIM (similarity) value of 13.5%, 9.4%, 7.6% and 5.9%, respectively.

The results of antibiotic sensitivity analysis are shown in Table 2. Strain H27 was sensitive to ceftriaxone, amoxicillin, streptomycin, gentamicin, clarithromycin, azithromycin, norfloxacin, ofloxacin, cotrimoxazole, chloramphenicol, florfenicol and polymyxin, and resistant to cefalexin, penicillin, kanamycin, tetracycline and furazolidone.

### 3.7. Bacterial Challenge Experiment of Larval C. gigas Using Strain H27

**Figure 7:**
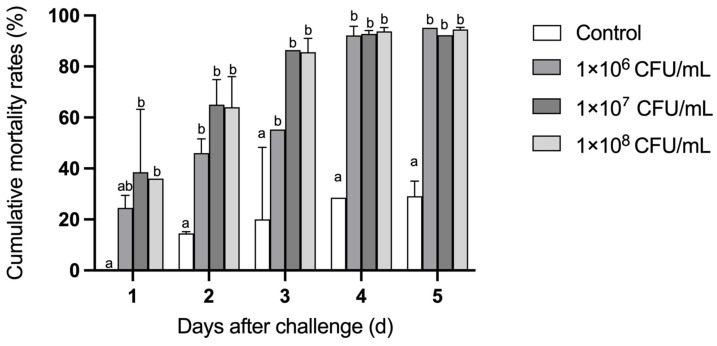
The survival rate of *C. gigas* larvae infected with strain H27 (*p* < 0.01). *C. gigas* larvae were challenged using the dipping method with sterile seawater (control group) and different concentrations of strain H27 (1 × 10^6^ CFU/mL, 1 × 10^7^ CFU/mL, 1 × 10^8^ CFU/mL). Survival rates were recorded daily for 5 days. Different alphabets on top of the columns mean significant difference.

**Figure 8:**
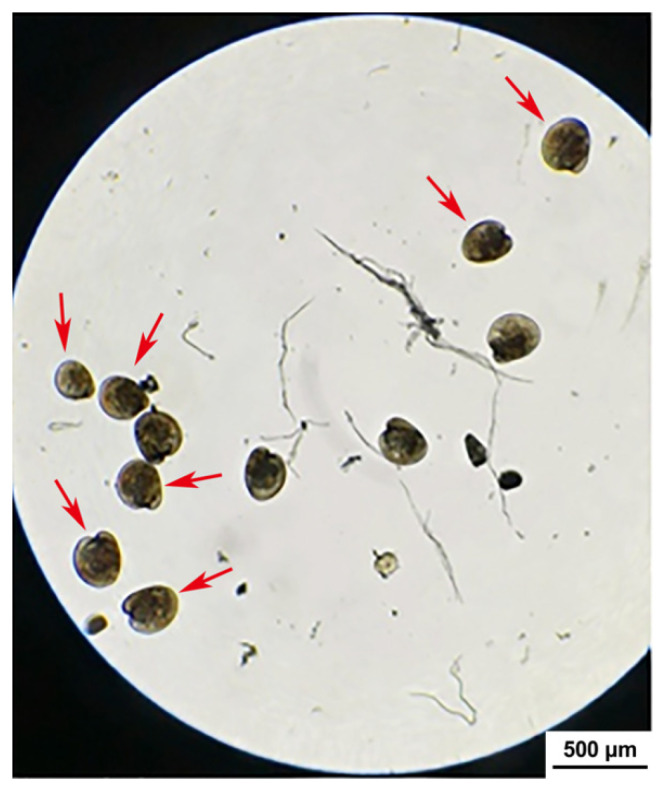
Microscopic image of *C. gigas* larvae challenged with strain H27 via immersion on day 2 (magnification: 100×). Reddened tissues are indicated using red arrows.

As shown in Figure 7, the mortality of the *C. gigas* larvae challenged with strain H27 was significantly higher than that of the larvae in the control group in the 5-day-experimental period (*p* < 0.01). In terms of the final mortality of the larvae challenged with strain H27 at the concentrations of 1 × 10^6^ CFU/mL, 1 × 10^7^ CFU/mL and 1 × 10^8^ CFU/mL, there was no significant difference between these three groups. After 1 day of the bacterial challenge experiment, mass death was observed in both of the two groups treated with higher concentrations of strain H27, with a mortality of nearly 40%. After 3 days of the bacterial challenge experiment, the mortality of both groups of larvae treated with higher concentrations of strain H27 was over 80%. In terms of the survival rate of the larvae challenged with strain H27 at the concentration of 1 × 10^6^ CFU/mL, it maintained a gradual decline in the first three days after infection. However, starting from day 4, the survival rate of the larvae dramatically decreased to a level which was comparable to that of the other two groups challenged with higher concentrations of strain H27. When observed under the microscope, the challenged larvae in the three treatment groups displayed weakened vitality and reddened tissues, as shown in Figure 8. Strain H27 was re-isolated from tissues of the moribund *C. gigas* larvae 3 days after the bacterial challenge, indicating its fulfillment of Koch’s postulate.

### 3.8. Histopathological Analysis of Strain H27-Challenged C. gigas Adults

**Figure 9:**
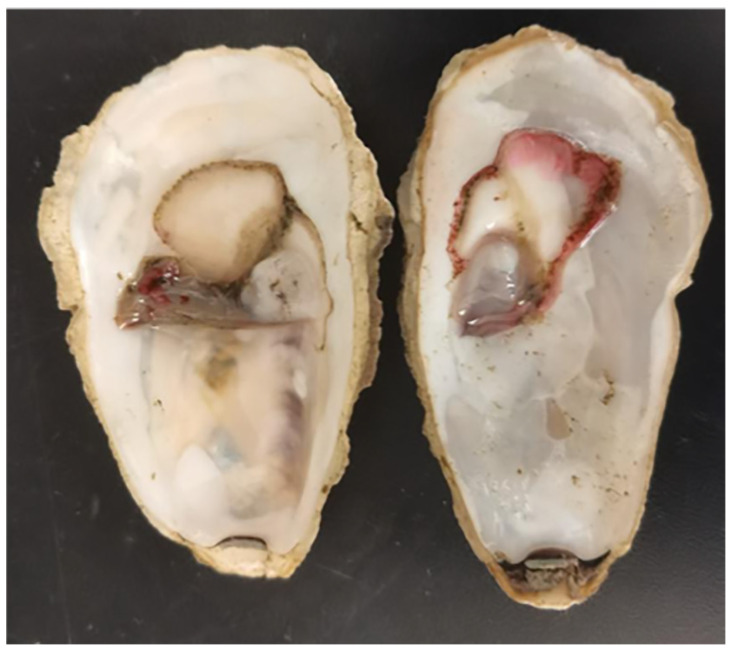
Adult *C. gigas* challenged with strain H27 via injection. It displayed a reddened mantle.

As shown in Figure 9, the moribund *C. gigas* displayed a reddened mantle. Additionally, the infected *C. gigas* showed decreased feeding ability.

**Figure 10:**
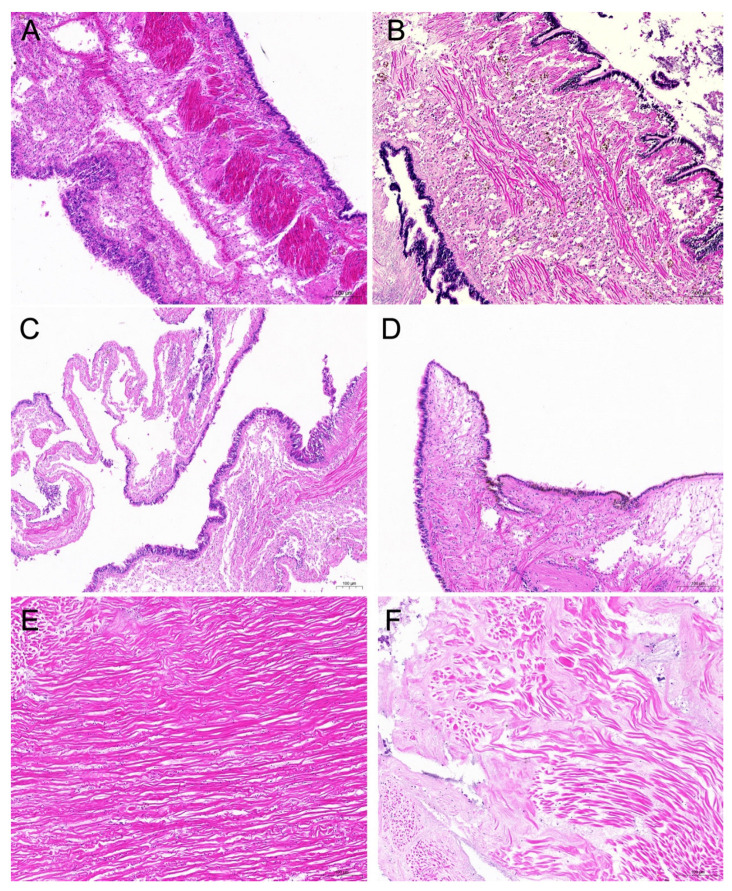
Histopathological analysis of the mantle (**A**–**D**) and adductor muscles (**E**,**F**) of adult *C. gigas*. (**A**,**C**,**E**): tissues of *C. gigas* in the control group. (**B**,**D**,**F**): tissues of *C. gigas* challenged with strain H27. A large number of yellowish-brown pigment granules were observed in the cytoplasm of the mantle. The connective tissue was broken and the epithelial cells were necrotic and scattered.

Histopathological analysis showed lesions in the mantle and adductor muscle of the diseased *C. gigas* (Figure 10). In the samples of mantle tissue, a large number of yellowish-brown pigment granules were observed in the cytoplasm of the mantle. The connective tissue was broken and the epithelial cells were necrotic and scattered (Figure 10B,D). Compared with the control group, the structure of the adductor muscle fibers of the diseased *C. gigas* was scattered and deformed, and the distance between the muscle fibers became larger (Figure 10F).

## 4. Discussion

*Crassostrea gigas* is a bivalve mollusk that inhabits estuaries and coasts [41]. As a filter feeder, it tends to accommodate a large number of microorganisms from the aquatic environment, including pathogenic bacteria. These pathogenic bacteria are mainly opportunistic and under suitable environmental conditions, they can quickly proliferate and then invade *C. gigas*, especially the larvae, leading to disease outbreak and even mass death. Therefore, the isolation and identification of pathogenic bacterial strains is of great significance to prevent and control *C. gigas* diseases. Its biochemical properties were in accordance with previously reported strains of the genus *Pseudoalteromonas* [11,31]. However, its metabolic profiles were not completely identical to any of the reported *Pseudoalteromonas* strains, which could be due to species-specific differences. The Biolog Microbial Identification System can automatically identify bacterial strains to the species level, given a SIM value of ≥50%. The highest SIM value generated by aligning the Biolog Gen III profiles of strain H27 to the existing database was as low as 13.5%, which was insufficient to support a species-level identification. The limited use of the Biolog Gen III database in accurate identification of environmental and marine bacteria has been reported in a previous study, where 24 out of 64 bacterial strains isolated from saline environments were identified to the species level [41]. In addition, due to variations in laboratory cultivation conditions for bacteria, the individual experiments may not be repeatable [42]. Therefore, in this study, strain H27 was only identified to the genus level through 16S rRNA gene sequencing and it was found to be most similar to *Pseudoalteromonas* sp. S49 (GenBank accession no.MN889221.1).

According to previous findings, bacterial pathogens of bivalve larvae mainly include the genera of *Vibrio*, *Pseudomonas*, *Alteromonas*, *Moraxella* and *Aeromonas* [43,44]. The genus *Pseudoalteromonas* was first proposed in 1995 [12]. Being a newly established marine bacterial genus, it is rarely directly connected with bivalve diseases. However, members of the *Pseudoalteromonas* genus could act as pathogens to other marine organisms. *P. agarivorans* NW4327 was reported to be pathogenic to sponge (*Rhopaloeides odorabile*) [45]. *P. shioyasakiensis* caused high mortality of juvenile abalone [46]. *P. piscicida* was isolated from fish suffering from Favobacteriosis [29]. There is also indirect evidence which connects *Pseudoalteromonas* with the outbreak of mariculture diseases. In 2015, *Pseudoalteromonas* was isolated as the dominant genus from diseased *Argopecten purpuratus* tissues in a scallop larvae hatchery located in Chile, accounting for 22.6% of total bacterial genera [47]. During mass death events of oysters, *Pseudoalteromonas* is also often present as a dominant genus in diseased oysters [33,34,48]. Consistent with previous findings, in this study, *Pseudoalteromonas* was the second most abundant genus in infected *C. gigas*, with a relatively higher abundance as compared with that of healthy *C. gigas* (with a concurrent upregulation of *Pseudoalteromonas* from 4.8% to 8.7%). Additionally, bacterial community analysis revealed a downregulation in the relative abundance of *Vibrio* from 54.9% in the healthy group to 30.6% in the infected group. This might be attributed to the colonization of opportunistic pathogenic *Pseudoalteromonas*. Previous findings have shown that species of *Pseudoalteromonas* showed moderate virulence to *C. gigas*, leading to a mortality of 30–40% [48]. When challenged with *P. shioyasakiensis* at a bacterial dose of 1 × 10^4^ CFU/mL, all *Haliotis discus hannai* died within 3 days [46]. In comparison with previous reports, the virulence of *P*. sp. H27 could possibly be at an intermediate level.

When inoculating strain H27 back to healthy *C. gigas* larvae and adults, it caused death and disease, reproducing the clinical signs originally observed. Upon histopathologic analysis, a large number of brown granules in the cytoplasm were observed in the mantle samples of challenged *C. gigas*. In addition, foci of tissue lysis were observed in the adductor muscle samples of challenged *C. gigas*. It has been established that brown cell proliferation and foci of tissue lysis are viewed as physiological, perhaps immunological coping mechanisms of bivalve mollusks against environmental stresses, infection with major pathogens, or concurrent secondary infections [49]. In a *C. gigas* disease caused by ostreid herpesvirus-1, these two clinical signs were observed [50]. In this study, brown cell proliferation and foci of tissue lysis were both observed in strain H27-challenged *C. gigas*, suggesting that strain H27 could be the causative agent of *C. gigas* disease.

With an objective of preventing and controlling the disease caused by this bacterium, the antibiotic sensitivity of *P.* sp. H27 was examined. Ceftriaxone, amoxicillin, streptomycin, gentamicin, clarithromycin, azithromycin, norfloxacin, ofloxacin, cotrimoxazole, chloramphenicol, florfenicol and polymyxin B could inhibit its growth, whereas cefalexin, penicillin, kanamycin, tetracycline and furazolidone could not. Our findings are roughly in accordance with existing reports and could provide reference for *C. gigas* disease control [17,29,30,51]. Therefore, for treatments using antimicrobial agents, drug sensitivity analysis of pathogenic bacterial strains should be taken into consideration. Antibiotics such as ceftriaxone, amoxicillin and streptomycin can be used to control *Pseudoalteromonas* infections. Moreover, to avoid drug resistance, bacterial infections should be prevented using probiotics. Additionally, monitoring environmental conditions and water quality may help to prevent and control diseases caused by *Pseudoalteromonas*.

*Pseudoalteromonas* is widely distributed in marine environments. As reported, some species produce extracellular enzymes, including agarase, β-galactosidase, α-amylase and pectin lyase [52]. Some produce antimicrobial metabolites (alkaloids, polyketides and peptides) and form a microbial barrier to protect the host from pathogen invasion, sometimes favoring the growth of bivalves [53]. Our results are the opposite to exisiting reports. Strain H27 was isolated from diseased *C. gigas* and was pathogenic to *C. gigas*, which not only demonstrated the functional versatility of the genus *Pseudoalteromonas*, but also enriched the knowledge on *Pseudoalteromonas* of marine environment origin. Environment greatly influences the physiological and biochemical characteristics of microorganisms. Previous findings have shown that *Pseudoalteromonas* was an opportunistic pathogen and was more likely to cause host diseases under favorable environmental conditions [46], such as high temperature and heavy rainfall [41,54]. The climatological data showed that in May 2023, Zhanjiang experienced relatively higher temperature and stronger rainfall, which might favor the propagation and pathogenicity of *P.* sp. H27. Both the virulence of bacteria and the immune responses of the host are largely affected by changes in physical environmental parameters, though the underlying mechanisms remain unclear [55]. In the future, further studies are required to elucidate the complex interactions among hosts, pathogens and the environment.

## 5. Conclusions

In this study, *Pseudoalteromonas* sp. H27, a bacterial strain pathogenic to *C. gigas*, was isolated from the diseased tissue of *C. gigas*, which could reproduce the originally observed clinical signs when challenging adult *C. gigas*. However, the specific pathogenesis has not yet been elucidated, which requires further research. This study provides valuable information for the infection of *Pseudoalteromonas* in *C. gigas*. From the perspective of the aquaculture industry, reducing pathogen loads and cultivating healthier *C. gigas* are of paramount importance. Maintaining optimal water quality, adjusting water temperatures and administering appropriate antibiotics to inhibit the growth of bacterial pathogens are feasible and cost-effective disease mitigation strategies. These strategies might contribute to the prevention and control of related diseases of *C. gigas*.

## Figures and Tables

**Table 1:** Metabolic profiles of strain H27 using Biolog Gen III Microplate.

	Reaction Item	Result		Reaction Item	Result
A1	Negative Control	-	E1	Gelatin	+
A2	Dextrin	+	E2	Glycyl-L-Proline	+
A3	D-Maltose	+	E3	L-Alanine	+
A4	D-Trehalose	-	E4	L-Arginine	+
A5	D-Cellobiose	-	E5	L-Aspartic Acid	-
A6	Gentiobiose	-	E6	L-Glutamic Acid	+
A7	Sucrose	-	E7	L-Histidine	-
A8	D-Turanose	-	E8	L-Pyroglutamic Acid	-
A9	Stachyose	-	E9	L-Serine	+
A10	Positive Control	+	E10	Lincomycin	-
A11	pH 6	+	E11	Guanidine HCl	-
A12	pH 5	-	E12	Niaproof4	-
B1	D-Raffinose	-	F1	Pectin	-
B2	α-D-Lactose	-	F2	D-Galacturonic Acid	+
B3	D-Melibiose	-	F3	L-Galactonic Acid Lactone	+
B4	β-Methyl-D-Glucoside	-	F4	D-Gluconic Acid	+
B5	D-Salicin	-	F5	D-Glucuronic Acid	+
B6	N-Acetyl-D-Glucosamine	+	F6	Glucuronamide	+
B7	N-Acetyl-β-D-Mannosamine	+	F7	Mucic Acid	-
B8	N-Acetyl-D-Galactosamine	-	F8	Quinic Acid	-
B9	N-Acetyl Neuraminic Acid	+	F9	D-Saccharic Acid	-
B10	1%NaCl	+	F10	Vancomycin	+
B11	4%NaCl	+	F11	Tetrezolium Violet	+
B12	8%NaCl	+	F12	Tetrezolium Blue	-
C1	α-D-Glucose	+	G1	p-Hydroxy-Phenylacetic Acid	-
C2	D-Mannose	-	G2	Methyl Pyruvate	-
C3	D-Fructose	+	G3	D-Lactic Acid Methyl Ester	-
C4	D-Galactose	+	G4	L-Lactic Acid	-
C5	3-Methyl Glucose	-	G5	Citric Acid	-
C6	D-Fucose	-	G6	α-Keto-Glutaric Acid	-
C7	L-Fucose	-	G7	D-Malic Acid	-
C8	L-Rhamnose	-	G8	L-Malic Acid	+
C9	Inosine	+	G9	Bromo-Succinic Acid	-
C10	1% Sodium Lactate	+	G10	Nalidixic Acid	-
C11	Fusidic Acid	+	G11	Lithium Chloride	-
C12	D-Serine	-	G12	Potassium Tellurite	+
D1	D-Sorbitol	-	H1	Tween 40	-
D2	D-Mannitol	+	H2	γ-Amino-Butyric Acid	-
D3	D-Arabitol	-	H3	α-Hydroxy-Butyric Acid	-
D4	myo-Inositol	-	H4	β-Hydroxy-D,L-butyric Acid	-
D5	Glycerol	-	H5	α-Keto-Butyric Acid	-
D6	D-Glucose-6-PO4	+	H6	Acetoacetic Acid	+
D7	D-Fructose-6-PO4	+	H7	Propionic Acid	+
D8	D-Aapartic Acid	-	H8	Acetic Acid	+
D9	D-Serine	-	H9	Formic Acid	-
D10	Troleandomycin	-	H10	Aztreonam	+
D11	Rifamycin SV	-	H11	Sodium Butyrate	-
D12	Minocycline	-	H12	Sodium Bromate	-

Notes: “+” means positive and “-” means negative. Negative control: A1; positive control: A10; carbon source utilization: 1–9; chemical sensitivity: D,E,F,G,H10–12 and environmental factors: A,B,C10–12.

**Table 2:** Drug sensitivity test results of strain H27.

Type of Antibiotic	Name of Antibiotic	Inhibitory Zone Diameter (mm)			Inhibitory Zone Diameter of H27 (mm)	Sensitivity
		R	I	S		
β-lactams	Cefalexin	≤14	15–17	≥18	0	R
	Ceftriaxone	≤13	20–22	≥23	25.73 ± 0.40	S
	Penicillin	≤19		≥20	13.23 ± 0.45	R
	Amoxicillin	≤13	14–17	≥18	20.90 ± 1.32	S
Aminoglycosides	Neomycin	≤12	13–16	≥17	15.43 ± 2.72	I
	Kanamycin	≤13	14–17	≥18	7.83 ± 0.90	R
	Streptomycin	≤11	12–14	≥15	16.53 ± 0.32	S
	Gentamicin	≤12	13–14	≥15	31.23 ± 1.50	S
Macrolides	Erythromycin	≤13	14–22	≥23	18.20 ± 1.15	I
	Clarithromycin	≤13	14–17	≥18	18.73 ± 0.67	S
	Azithromycin	≤13	14–17	≥18	21.63 ± 2.87	S
Quinolones	Norfloxacin	≤12	13–16	≥17	24.93 ± 0.58	S
	Ofloxacin	≤12	13–15	≥16	21.13 ± 0.12	S
Sulfonamides	Cotrimoxazole	≤10	11–15	≥16	19.53 ± 1.26	S
Tetracyclines	Tetracyclines	≤14	15–18	≥19	7.87 ± 0.50	R
Chloramphenicols	Chloromycetin	≤12	13–17	≥18	26.63 ± 2.33	S
	Florfenicol	≤12	13–17	≥18	25.90 ± 0.44	S
Polypeptides	PolymyxinB	≤8	9–11	≥12	17.73 ± 1.14	S
Nitrofurans	Furazolidone	≤14	15–16	≥17	12.93 ± 2.58	R

Notes: “R” represents resistant; “I” represents intermediate; “S” represents sensitive.

## Data Availability

The original contributions presented in this study are included in the article. Further inquiries can be directed to the corresponding authors.
